# Perspective-dependent reactivity of sensorimotor mu rhythm in alpha and beta ranges during action observation: an EEG study

**DOI:** 10.1038/s41598-018-30912-w

**Published:** 2018-08-20

**Authors:** Monica Angelini, Maddalena Fabbri-Destro, Nicola Francesco Lopomo, Massimiliano Gobbo, Giacomo Rizzolatti, Pietro Avanzini

**Affiliations:** 1 Consiglio Nazionale delle Ricerche (CNR), Istituto di Neuroscienze, Sede di Parma, Italy; 2 Dipartimento di Ingegneria dell’Informazione, Università degli Studi di Brescia, Brescia, Italy; 3 Dipartimento di Scienze Cliniche e Sperimentali, Università degli Studi di Brescia, Brescia, Italy

## Abstract

During action observation, several visual features of observed actions can modulate the level of sensorimotor reactivity in the onlooker. Among possibly relevant parameters, one of the less investigated in humans is the visual perspective from which actions are observed. In the present EEG study, we assessed the reactivity of alpha and beta mu rhythm subcomponents to four different visual perspectives, defined by the position of the observer relative to the moving agent (identifying first-person, third-person and lateral viewpoints) and by the anatomical compatibility of observed effectors with self- or other individual’s body (identifying ego- and allo-centric viewpoints, respectively). Overall, the strongest sensorimotor responsiveness emerged for first-person perspective. Furthermore, we found different patterns of perspective-dependent reactivity in rolandic alpha and beta ranges, with the former tuned to visuospatial details of observed actions and the latter tuned to action-related parameters (such as the direction of actions relative to the observer), suggesting a higher recruitment of beta motor rhythm in face-to-face interactions. The impact of these findings on the selection of most effective action stimuli for “Action Observation Treatment” neurorehabilitative protocols is discussed.

## Introduction

To date, ample evidence suggests that the observation of actions performed by another individual can automatically activate cortical motor networks similar to those involved in the actual performance of the observed action, through the so-called Mirror Neurons System (MNS)^1–3^. This direct matching of the viewed actions with their motor counterparts in observer’s brain would represent the neural substrate not only for movement imitation, but also for higher order social processes such as observational learning and action understanding^1–3^. Recent lines of research pointed out that several visual features of observed actions can influence the level of activation of MNS and the consequent priming of the motor system during action observation (AO) (for review, see^3^). In this regard, among different parameters of observed actions, the visual perspective from which a movement is seen remains underinvestigated in humans, and, consequently, conclusive data about the modulatory effect of different viewpoints on the motor system reactivity during AO are still lacking.

Indeed, the AO perspectives can be described in two main systems of coordinates. The first, Cartesian, defines the spatial relationship between the observer and the moving agent in terms of distances and angles. Accordingly, perspectives can be defined as longitudinal or lateral. Longitudinal include the first-person perspective (FP), the natural viewpoint from which self-actions are usually observed, and third-person perspective (TP), with the observer facing the moving agent. Lateral perspectives include viewpoints in which the observer looks at the moving agent from a lateral position. The second system of coordinates, body centred, defines the anatomical compatibility of the observed moving effectors with self-body parts. Accordingly, perspectives can be defined as egocentric or allocentric. In egocentric perspectives, observed actions are morphologically consistent with those seen during movements of one’s own body segments. Conversely, in allocentric perspectives visual information is anatomically congruent only with the body parts of another individual.

Combining these coordinates systems, both longitudinal and lateral perspectives can be labelled as either ego- or allo-centric. Indeed, FP and TP correspond to longitudinal ego- and allo-centric viewpoints, respectively. Furthermore, ego- and allo-centric points of view could be assumed also for lateral perspectives. For instance, seeing a right hand from the right side of a moving agent represents a lateral allocentric perspective (LA), since this viewpoint precludes a direct spatial and morphological matching with the view of body parts of the observer. Conversely, seeing a right hand from the left side of a moving agent represents a lateral egocentric perspective (LE), since visual information is anatomically compatible with the observer’s body.

A still open question in AO literature is which perspective is the most effective in evoking motor resonance in the observer. Theoretically, others’ actions perceived from egocentric perspectives, being more visually similar to self-actions, should induce a stronger activation in the motor system of the observer with respect to actions perceived from allocentric viewpoints. Nevertheless, experimental data so far available in monkeys^4–6^ and humans^7–15^ are inconsistent and definite evidence is still lacking. Indeed, a single-cell recording study in macaques revealed view-dependent responses in most of F5 mirror neurons (MNs), which were tuned to one or, more frequently, two viewpoints among three tested perspectives (FP, lateral and TP). Although a higher percentage of MNs responded to FP, a significant preference for one of the three viewpoints did not emerge^4^. Nonetheless, a further study on local field potentials (LFPs) of F5 MNs confirmed the preference for FP relative to TP^5^. More recently, single-cell recordings demonstrated a prevalent tuning of F5 MNs to FP than to LE during observation of actions carried out in monkey’s peripersonal space^6^. While AO experiments in monkeys included both longitudinal and lateral perspectives, human studies compared only cerebral responses evoked by FP vs TP, with inconsistent results. Different transcranial magnetic stimulation studies reported larger motor evoked potentials (MEPs) during observation of either egocentric^7^ or allocentric^8^ movements, while others reported no difference in MEPs between these perspectives^9^. Some functional magnetic resonance imaging (fMRI) studies^10,11^ found stronger activity in sensorimotor cortex during AO from FP relative to TP. In contrast, other authors reported only visual differences in occipital areas for the FP vs TP comparison, whereas motor-related areas resulted perspective invariant^12^. Inconsistent results also emerged in electroencephalographic (EEG) studies, which tested the effect of FP and TP on the motor system by assessing modulations of central sensorimotor mu rhythm during AO. Mu rhythm is an oscillatory activity recorded from central electrodes overlying primary sensorimotor cortex and includes at least two nonharmonic components, in alpha (8–13 Hz) and beta (14–25 Hz) frequency bands^16,17^. Different source locations and reactivity during various motor tasks have been revealed for the two components^16–19^, suggesting their different functional roles. The alpha subcomponent would reflect a predominant sensorimotor function, while the beta component would be more closely associated with motor cortical control. Overall, mu suppression during voluntary movements would index the activation of sensorimotor cortex, subtended by asynchronous firing pattern of sensorimotor neurons. Similarly, mu desynchronization during AO would reflect modulation of sensorimotor cortex by the activity of MNS^20^. On the one hand, stronger desynchronization of alpha mu subcomponent during observation of hand actions from TP compared to FP has been reported^13^. This result has been interpreted as reflecting higher processing load required by TP to match perceived movements with the observer’s egocentric frame of reference. On the other hand, further EEG studies that showed opposite results^14,15^ pointed to a closer link with the sensorimotor system for FP, due to a simplified matching of movement parameters. To date, evidence in humans about the reactivity of the beta mu subcomponent to different AO viewpoints, as well as about modulatory effects of lateral with respect to frontal perspectives on mu rhythm is still lacking.

Of note the definition of the perspective that triggers the strongest level of motor reactivity in the onlooker during AO, has not only theoretical implications in the field of motor cognition^1,2^, but also practical applications. Indeed, AO has been exploited in a novel rehabilitation approach (the so-called Action Observation Treatment, AOT) as an adjunct to conventional physical therapy to promote motor recovery in neurological and non-neurological conditions (for reviews, see^21,22^). Given the potential application of AO as a neurorehabilitation tool, a key step to maximize motor recovery is to define the optimal visual parameters of the displayed actions that, strategically manipulated, can lead to the strongest motor resonance in the observer’s brain. So far, AOT protocols displayed action stimuli from several perspectives, ranging from studies using only the FP^23–25^ to studies including different combinations of FP, TP and lateral viewpoints^26–28^. Nevertheless, the choice of such points of view was empirical, since conclusive evidence about which perspective is the most effective in priming the motor system is still lacking.

Given this theoretical background, we performed a high-density EEG study, comparing the modulation in alpha and beta mu rhythm subcomponents induced by the observation of actions from four different perspectives. We evaluated EEG responses to observation of actions performed by both a right and a left hand, taking into account not only FP and TP, but also lateral ego- and allo-centric viewpoints. Through the assessment of mu rhythm modulation during AO, the results of the present study may lead to the selection of the most appropriate, neurophysiologically-grounded stimuli applicable in future AOT rehabilitative protocols.

## Methods

### Participants

Sixteen healthy Caucasian volunteers (nine male) took part in the study; mean age (standard deviation) (SD) was 26.12 (3.89) years. All participants had normal or corrected-to-normal visual acuity and were right handed, as assessed by the Edinburgh Handedness Inventory^29^. None of them reported anamnestically previous or actual neurological or psychiatric disorders or the use of chronic pharmacological treatment. Before the experiment, participants gave their written informed consent to the study, which was approved by the local ethical committee (Comitato Etico Unico per la Provincia di Parma) and was conducted in accordance with the principles expressed in the Declaration of Helsinki.

### Stimuli

**Figure 1:**
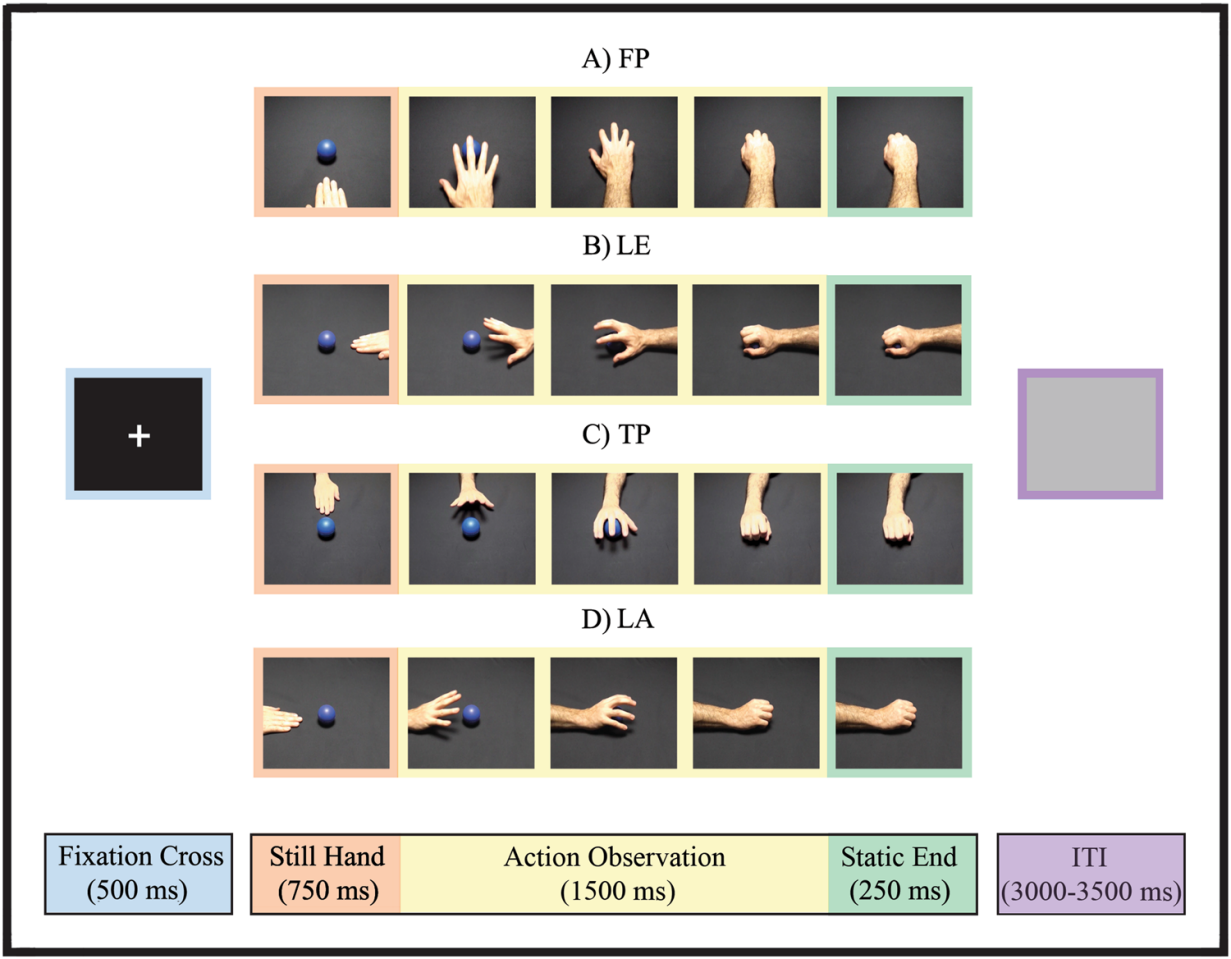
Experimental stimuli and structure of the Condition Trials. Trial events, their durations and single frames extracted from video clips of each perspective are shown. As an example of experimental stimuli, the grasping movement performed with the right hand by the male actor is displayed, from each of the four perspectives. FP: first-person perspective; LE: lateral egocentric perspective; TP: third-person perspective; LA: lateral allocentric perspective.

Experimental stimuli consisted of video clips (each 2500 ms long) showing one of two possible reach-to-grasp actions, ending with either a precision grip or a whole-hand grasp of a target object (a blue ball, diameter 6 cm) (Fig. 1, Supplementary Fig. S1). The actions were performed with the right or left hand (RH, LH, respectively) by two actors (one male, one female) and were presented from four different points of view: FP, TP, LA, LE.

As stimuli were administered through video clips, it is relevant to verify how stimuli with different perspectives projected onto the visual field, i.e., the video frames. The trajectory of the actions caught from longitudinal and lateral perspectives developed along two orthogonal axes, intersecting in the centre of the screen. As a convention and from here on, we set the FP at 0° and, rotating counterclockwise, the TP at 180°. The angle of lateral perspectives varied according to the observed hand: for LE, RH was at 90° and LH and −90°; the reverse occurred for LA.

Each video clip showed in sequence: (1) the target object and the actor’s forearm in a resting position (duration: 750 ms); (2) the reach-to-grasp action (duration: 1500 ms); (3) the hand statically holding the target at the final position (duration: 250 ms). Thirty-two different video clips were recorded and then subdivided in eight different experimental conditions, defined by the combination of four Perspectives (FP, LA, LE, TP) and two Observed Hands (LH, RH): (1) FP-LH; (2) FP-RH; (3) LA-LH; (4) LA-RH; (5) LE-LH; (6) LE-RH; (7) TP-LH; (8) TP-RH. In half of the video clips the action was performed by the male and in the other half by the female actor. To avoid differences in action kinematics, the same action was simultaneously recorded by four camcorders placed one in front of the other, at a fix height approximately corresponding to the actors’ eyes, with a viewing angle of about 45° with the horizontal plane and on the same line of the actor’s resting hand and/or of the target object. Visual stimuli were administered using E-Prime software (Psychology Software Tools. Inc).

### Procedure

Participants sat at a distance of 60 cm in front of a 19-inches LCD computer screen, with their hands lying still on a keyboard and the index fingers placed relaxed on two specific keys (see below). They were asked to observe the video stimuli of the eight experimental conditions described above.

In each Condition Trial (Fig. 1), the following elements were presented in sequence: (1) a central fixation cross (duration: 500 ms); (2) the video clip (duration: 2500 ms); (3) an intertrial interval (ITI) (duration randomly variable between 3000 and 3500 ms). The experimental procedure included 320 trials (40 trials for each condition), which were randomly presented in five blocks. To ensure participants’ attention, 32 Catch Trials were randomly presented (from five to eight for each block), in which the video clip showed the movement stopping at different timings preceding the hand-object contact. Randomly after half of the blocked video clips, a question (“Completed?” or “Blocked?”) appeared on the screen (duration: 3000 ms). Participants had to respond whether in the last video clip the movement was completed or blocked, by pressing two different keys on a keyboard, with the right or left index finger that were placed relaxed on them. Half of Catch Trials required an affirmative answer, half a negative one. The finger (left or right)/response (“Yes” or “No”) matching was balanced across participants. Catch Trials were excluded from subsequent analyses.

Before starting the EEG recording, during a brief training phase, participants were randomly presented with 12 Condition Trials and 12 Catch Trials; they were asked to observe the video clips and were instructed on how to respond to the questions in Catch Trials.

To rule out muscular activation that could induce mu rhythm desynchronization during AO, surface electromyography (EMG) of first dorsal interosseous (FDI) muscles of participants’ hands was recorded (sampling rate 5000 Hz, bipolar derivation) during Condition Trials from 1000 ms of the ITI preceding the Fixation Cross until the end of the video clip. EMG data were bandpass filtered at 20–500 Hz and rectified. Afterwards, each EMG trial was visually inspected, blindly with respect to the concurrent EEG signal, with the aim to exclude all epochs presenting an EMG burst. Overall, an average (SD) of 9.8 (4.8) trials (3.06%) was rejected based on EMG, confirming the adherence of the participants to the experimental procedure requiring no movements during AO. EEG trials with concomitant EMG activity were excluded from further analyses (for further details, see “EMG Analyses and Results” in “Supplementary Information”).

### EEG Recording and analyses

Continuous EEG was recorded using the 128-channel Geodesic EEG System (Electrical Geodesics Inc., Oregon) and the HydroCel Geodesic Sensor Net (GSN300), at a sampling rate of 500 Hz (0.01 Hz high-pass filter) with the vertex as on-line reference; electrodes impedances were kept below 50 kΩ.

EEG data were filtered offline (1–35 Hz) and segmented in single-trial epoch lasting 4000 ms. Each epoch included: (1) 1000 ms of ITI screen preceding the fixation cross; (2) the fixation cross (500 ms); (3) the whole video (2500 ms). Trials with concomitant EMG activity were excluded from further analyses (see also “EEG Preprocessing” in “Supplementary Information”).

Off-line analyses were performed with MATLAB (The MathWorks, Inc., Natick, Massachusetts, United States) and EEGLAB toolbox^30^. EEG data were analysed by means of Independent Component Analysis, to remove components endowing ocular, cardiac or muscular artefacts, and further visually inspected to exclude other residual artefacts. Artefacted channels were interpolated and EEG data were re-referenced to the linked mastoids.

For each accepted and cleaned epoch, time-frequency transforms were computed for each electrode in the frequency range from 5 to 32 Hz (55 output frequencies, frequency resolution 0.5 Hz), by means of a Morlet wavelet with the number of cycles increasing linearly with frequency (from 4 cycles at the lowest to 15.36 cycles at the highest frequency)^30^. In order to minimize sensitivity to outlier and noisy trials, each single-trial time-frequency transform was normalized by dividing each frequency by the mean spectral power of the same frequency over the full-epoch length^31^. Afterward, a 300 ms prestimulus period within the ITI (from −1150 to −850 ms before video clip onset) served as a baseline, and for each participant and condition, averaged spectral data were baseline corrected by dividing each time-frequency point value by average spectral power of the baseline at the same frequency. The ratio was used to account for individual variability in overall EEG power. Resulting data were expressed as absolute power ratio with respect to baseline activity: an absolute ratio value of 1 indicated no difference relative to the baseline, values < 1 represented desynchronization (i.e., EEG power reduction) and values > 1 represented synchronization (i.e., EEG power increase).

**Figure 2:**
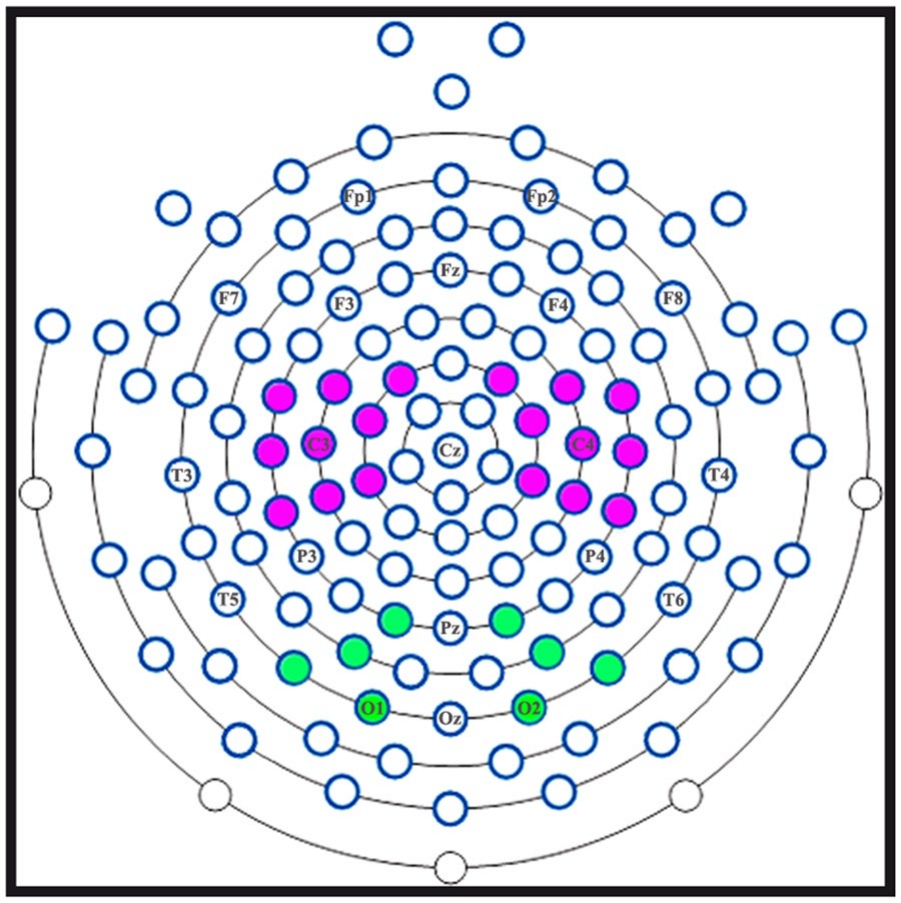
Clusters of electrodes used for EEG statistical analyses. Top view of the 128-channel EEG array, showing the two symmetrical central clusters of electrodes around standard C3 and C4 (purple circles) and the two symmetrical posterior clusters of electrodes around standard O1 and O2 (green circles).

**Figure 3:**
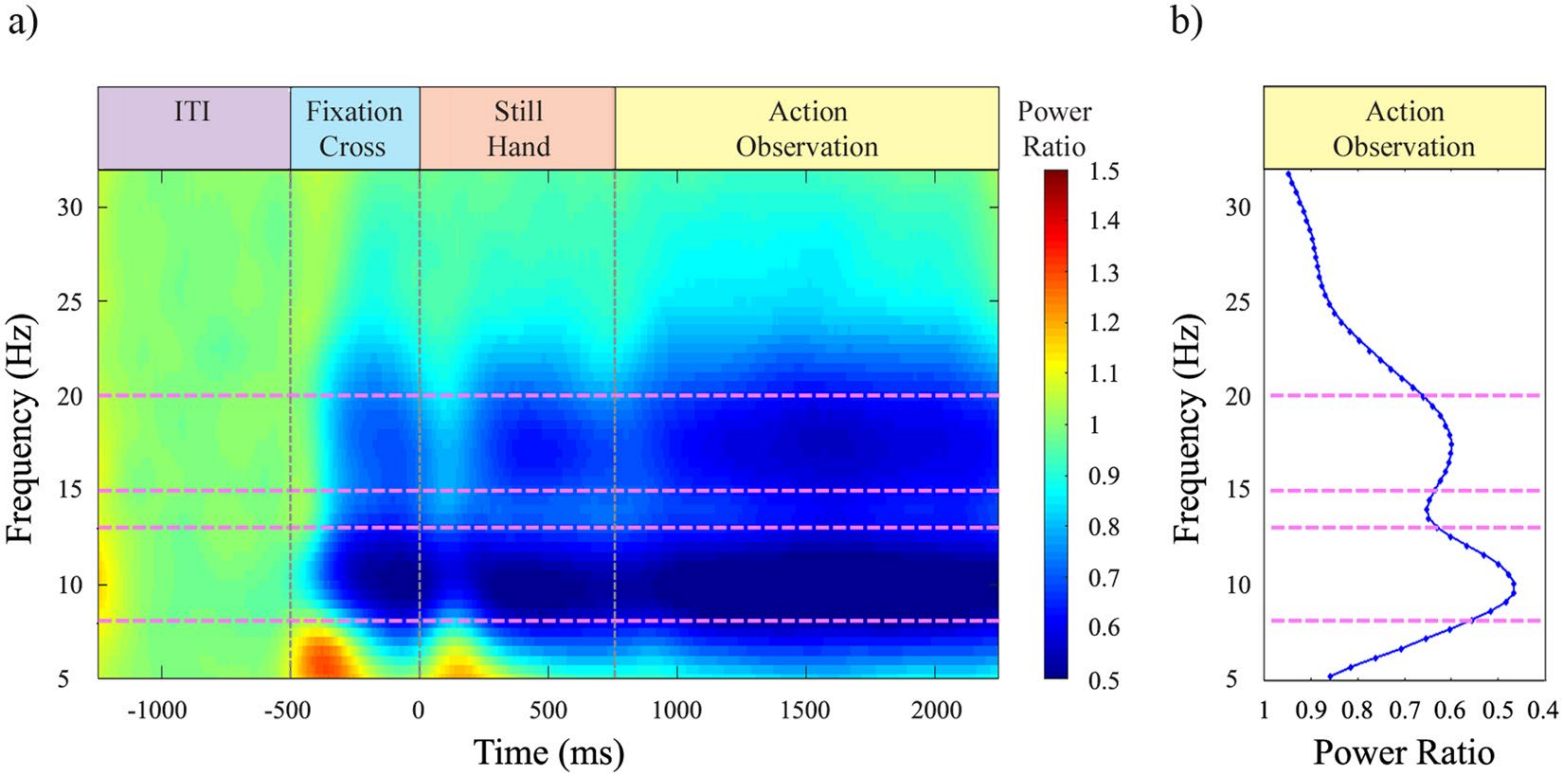
Grand-averaged power ratio, averaged across all experimental conditions and right and left central clusters of electrodes. **(a)** Time-frequency panel of grand-averaged absolute power ratio for the whole epoch. The onset of the video clip is at 0 ms. **(b)** Averaged absolute power ratio for each frequency during action observation period. Pink dashed lines indicate the analysed alpha (8–13 Hz) and beta (15–20 Hz) frequency ranges, around frequency peaks of maximum desynchronization during action observation period at 10 and 17.5 Hz.

Following previous literature (e.g.,^32,33^), mu rhythm analyses were focused on two perirolandic clusters of electrodes around C3 and C4 (Fig. 2), one for each hemisphere. Given evidence that mu rhythm subcomponents might have different functional properties^16,17,34^, to select frequency bands of interest in a data-driven manner, we averaged ratio data across participants, conditions and right and left central clusters of electrodes (Fig. 3a). Afterward, we averaged in time such data during the AO period. The resulting plot (Fig. 3b) showed two peaks of desynchronization during AO, at 10 and 17.5 Hz respectively, around which we centred the two frequency bands of interest for subsequent analyses, namely alpha (8–13 Hz) and beta (15–20 Hz) bands. Separately for each participant, cluster of electrodes, frequency band of interest and condition, ratio data were averaged in time in four different periods of interest within the epoch: (1) the ITI period; (2) the 500 ms corresponding to the Fixation Cross presentation; (3) the 750 ms corresponding to the Still Hand presentation in the initial part of video clips; and (4) the 1500 ms of AO (Fig. 3a). Furthermore, to render the temporal dynamics of mu rhythm modulation during AO, AO period was subdivided in six time windows (TWs), each lasting 250 ms. Due to inherent non-normality of ratio data, a log10 transform was calculated for each absolute power ratio value before statistical analyses^31^. A log10 ratio value of 0 indicated no disn, and positive log10 ratio EEG power increase. EEG statistical analyses followed a stepwise approach.

1. In a first step, we aimed at preliminarily testing whether all perspectives elicited a significant central visuomotor reactivity during AO. To do so, we evaluated for each perspective modulations of mu rhythm with respect to the baseline during whole AO period. Single-subject values were averaged across Observed Hand (LH, RH) and central left and right clusters of electrodes, separately for each perspective and frequency band. Log10 AO ratio data were tested in one-tailed single-sample *t* tests against 0 (*p < *0.05). For each perspective, log10 ratio values significantly lower than 0 indicated significant suppression of central alpha and beta components during AO relative to the baseline.
2. In a second step, we aimed at identifying AO TWs in which mu reactivity was modulated by AO and, at the same time, sensitive to the effect of perspective. To do so, on the one hand, we evaluated in which AO TW mu suppression was significantly stronger than in all the preceding periods of interest (namely ITI, Fixation Cross and Still Hand periods) for all perspectives. This allowed us to verify whether and when mu reactivity during AO was specific for the observation of biological movements with respect to different visual stimuli, which can induce mu modulations unrelated to AO and associated with spatial or attentional processing. On the other hand, we assessed whether and when the tested perspectives induced significant differences in mu suppression during AO. Ratio data were averaged across Observed Hands and right and left central cluster of electrodes, separately for each Perspective and frequency band. Log10 ratio values were tested in a repeated-measures ANOVA (*p* < 0.05) with Perspective (4 levels: FP, LA, LE, TP) and Time Window (9 levels: ITI, Fixation Cross and Still Hand periods, six TWs during AO) as within-subject factors. In case of significant Perspective × Time Window interaction, we computed post hoc tests and selected for further analyses AO TWs in which two criteria were satisfied: 1) all perspectives induced significantly stronger mu desynchronization with respect to all preceding periods of interest; and 2) a significant modulation of mu suppression across perspectives occurred (i.e., at least one significant comparison). Power ratio were averaged across those significant AO TWs and were further analysed in the following step.
3. In a third step, we aimed at assessing the influence of the two observed hands on mu suppression and the involvement of the two hemispheres, specifically in the AO TWs identified above. Power ratio data (averaged across significant AO TWs) were tested in a repeated-measure ANOVA (*p* < 0.05) with Perspective (4 levels: FP, LA, LE, TP), Observed Hand (2 levels: LH, RH) and Hemisphere (2 levels: central left and right clusters of electrodes) as within-subject factors, separately for each frequency range.

For all ANOVAs, in case of significant main effects and/or interactions, Bonferroni-corrected post hoc tests were computed and, in case of violation of the sphericity assumption at Mauchly’s test, Greenhouse-Geisser correction was applied to the degrees of freedom. For simplicity, uncorrected degrees of freedom are reported, together with corrected and significant *p* values.

### EEG Complementary analyses

In order to rule out the influence of volume conduction of concomitant posterior alpha rhythm on central alpha mu subcomponent, complementary analyses were performed on two symmetrical occipitoparietal clusters of electrodes around O1 and O2 (Fig. 2), one for each hemisphere. For further details, see “EEG Complementary Analyses” in “Supplementary Information”.

## Results

Results of the three steps of EEG statistical analyses are presented separately for alpha and beta mu subcomponents.

### Alpha mu subcomponent

#### Single-sample *t* tests

For all perspectives, one-tailed single-sample *t* tests against 0 resulted significant (FP *t*(15) = −10.62, LA *t*(15) = −11.98, LE *t*(15) = −14.04, TP *t*(15) = −11.09; all *p*s < 0.001), proving that during AO all perspectives induced an overall significant suppression of alpha mu subcomponent with respect to the baseline.

#### 4 (Perspective) × 9 (Time Window) repeated-measures ANOVA

**Figure 4:**
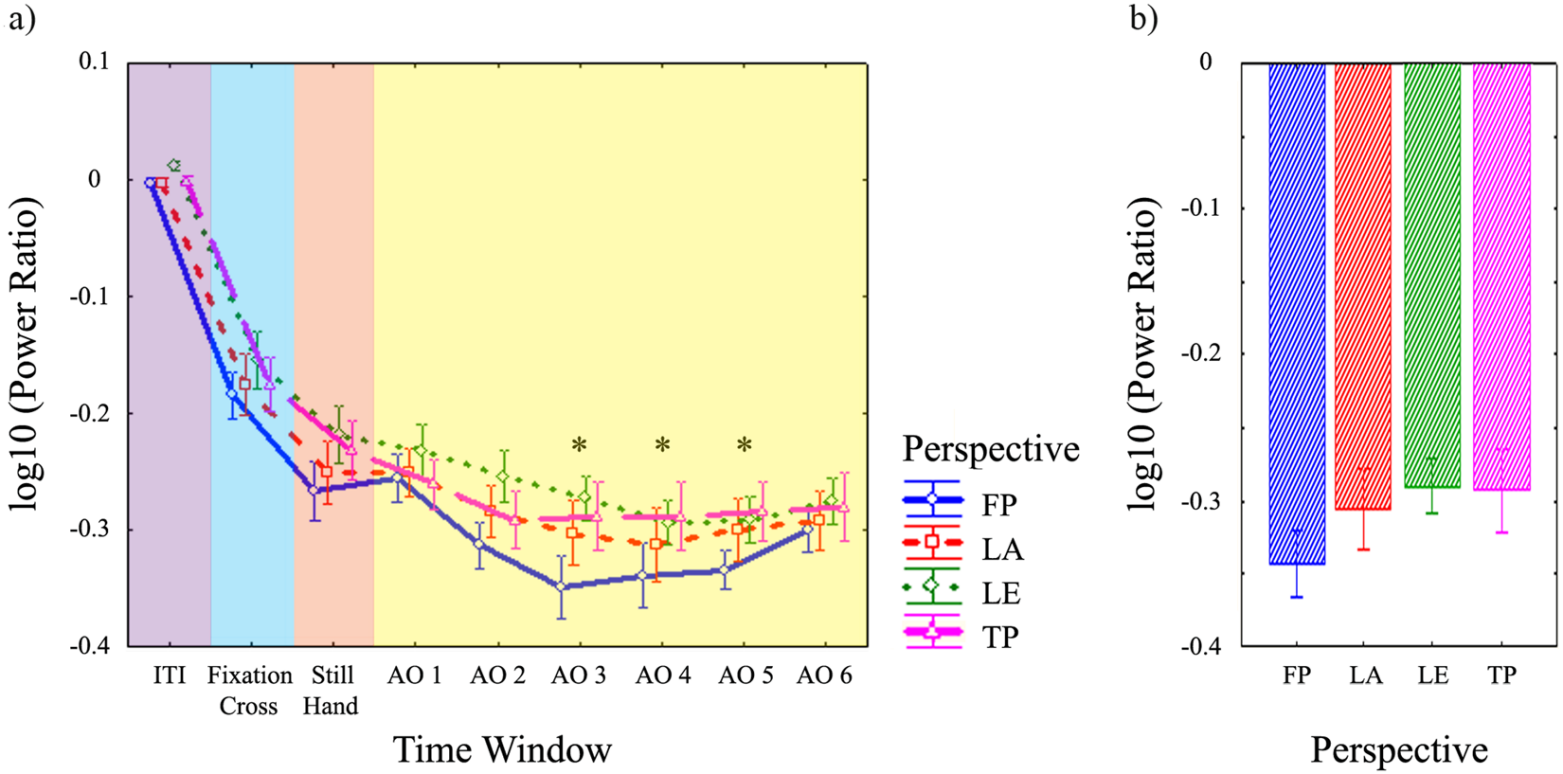
Results of EEG statistical analyses on power ratio data in alpha (8–13 Hz) frequency range from central clusters of electrodes. **(a)** Time course of alpha (8–13 Hz) log10 power ratio for each perspective, averaged across right and left central clusters of electrodes and left and right observed moving hands. The figure represents the significant Perspective × Time Window interaction of the 4 (Perspective) × 9 (Time Window) repeated-measures ANOVA performed on central alpha log10 power ratio. Each time window within the epoch (ITI, Fixation Cross, Still Hand presentation periods, six Action Observation time windows of 250 ms each) is labelled by different colours. Action observation time windows with both: 1) significantly stronger desynchronization for all perspectives with respect to ITI, Fixation Cross and Still Hand presentation periods; and 2) significant differences among perspectives, are indicated by asterisks. **(b)** Log10 power ratio of central alpha frequency range for each perspective, averaged during action observation time windows 3, 4, 5. The figure represents the significant main effect of Perspective in the 4 (Perspective) × 2 (Observed Hand) × 2 (Hemisphere) repeated-measures ANOVA performed on central log10 power ratio in the alpha range. Error bars represent the standard errors of the mean. ITI: intertrial interval period; AO: Action Observation time window. Other conventions as in Fig. 1.

The 4 (Perspective) × 9 (Time Window) repeated-measures ANOVA showed significant main effects of Perspective (*F*(3,45) = 3.88, *p* = 0.015, η_p_^2^ = 0.205) and Time Window (*F*(8,120) = 97.95, *p* < 0.001, η_p_^2^ = 0.867) and a significant Perspective × Time Window interaction (*F*(24,360) = 1.83, *p* = 0.01, η_p_^2^ = 0.109) (Fig. 4a).

For the main effect of Perspective, Bonferroni-corrected post hoc tests showed an overall stronger suppression of alpha mu subcomponent for FP than LE (*p* = 0.01). For the main effect of Time Window, post hoc tests showed a stronger suppression than in all preceding periods of interest during AO TWs 3, 4, 5 (Still Hand vs: AO TW3 *p* = 0.0012, AO TW5 *p* = 0.0014; all other *p*s < 0.001).

A significant Perspective × Time Window interaction modulated both main effects. Post hoc tests for the interaction confirmed that central alpha suppression during AO was stronger than during the three preceding periods of interest, simultaneously for all perspectives, in AO TWs 3, 4 and 5 (AO TW3 vs Still Hand: LA *p* = 0.014, LE *p* = 0.005, TP *p* = 0.002; AO TW4 vs Still Hand: TP *p* = 0.002; AO TW5 vs Still Hand: LA *p* = 0.037, TP *p* = 0.01; all other *p*s < 0.001). Furthermore, in these significant AO TWs, a modulation of suppression across perspectives emerged. During AO TW3, FP induced stronger desynchronization than LE and TP (both *p*s < 0.001) (with a trend toward significance vs LA, *p* = 0.08). During AO TWs 4 and 5, FP induced stronger desynchronization than TP (*p* = 0.02, *p* = 0.026, respectively).

In summary, both significantly stronger suppression of central alpha for AO than in preceding periods for all perspectives and significant modulation of suppression across perspectives, were present in AO TWs 3, 4 and 5, which were selected for further analyses.

#### 4 (Perspective) × 2 (Observed Hand) × 2 (Hemisphere) repeated-measures ANOVA

Given the results of the previous ANOVA, to evaluate the effect of the observed hand on mu suppression and the involvement of the two hemispheres in different AO perspectives, we averaged power ratio data over AO TWs 3, 4 and 5.

The 4 (Perspective) × 2 (Observed Hand) × 2 (Hemisphere) repeated-measures ANOVA showed significant main effects for Perspective (*F*(3,45) = 3.79, *p* = 0.017, η_p_^2^ = 0.202) and Hemisphere (*F*(1,15) = 5.16, *p* = 0.038, η_p_^2^ = 0.256). FP induced stronger alpha suppression than LE (*p* = 0.033) and TP (*p* = 0.034) (Fig. 4b). Furthermore, suppression was stronger in left than in right hemisphere.

### Beta mu subcomponent

#### Single-sample *t* tests

For all perspectives, one-tailed single-sample *t* tests against 0 resulted significant (FP *t*(15) = −8.85, LA *t*(15) = −8.75, LE *t*(15) = −9.41, TP *t*(15) = −7,53; all *p*s < 0.001), proving a significant suppression of beta mu subcomponent relative to the baseline during AO from all perspectives.

#### 4 (Perspective) × 9 (Time Window) repeated-measures ANOVA

**Figure 5:**
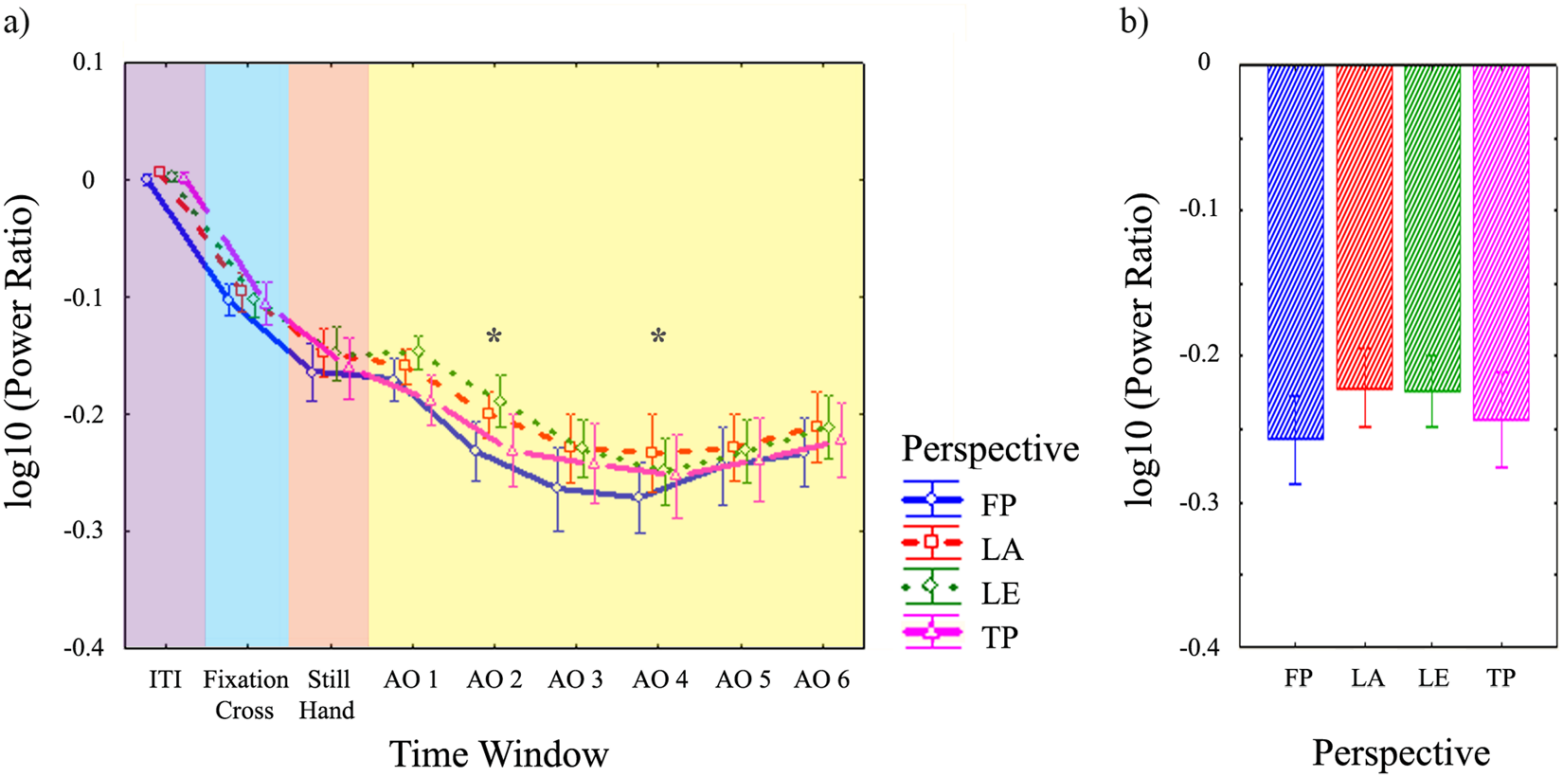
Results of EEG statistical analyses on power ratio data in beta (15–20 Hz) frequency range from central clusters of electrodes. **(a)** Time course of beta (15–20 Hz) log10 power ratio for each perspective, averaged across right and left central clusters of electrodes and left and right observed moving hands. The figure represents the significant Perspective × Time Window interaction of the 4 (Perspective) × 9 (Time Window) repeated-measures ANOVA performed on central beta log10 power ratio. Each time window within the epoch (ITI, Fixation Cross, Still Hand presentation periods, six Action Observation time windows of 250 ms each) is labelled by different colours. Action observation time windows with both 1) significantly stronger desynchronization for all perspectives with respect to ITI, Fixation Cross and Still Hand presentation periods; and 2) significant differences among perspectives, are indicated by asterisks. **(b)** Log10 power ratio of central beta frequency range for each perspective, averaged during action observation time windows 2, 3, 4. The figure represents the significant main effect of Perspective in the 4 (Perspective) × 2 (Observed Hand) × 2 (Hemisphere) repeated-measures ANOVA performed on central log10 power ratio in the beta range. All conventions as in Fig. 4.

The 4 (Perspective) × 9 (Time Window) repeated-measures ANOVA showed significant main effect of Time Window (*F*(8,120) = 45.21, *p* < 0.001, η_p_^2^ = 0.751) and Perspective × Time Window interaction (*F*(24,360) = 1.68, *p* = 0.024, η_p_^2^ = 0.101) (Fig. 5a).

For the main effect of Time Window, post hoc tests showed significantly stronger suppression than in preceding periods of interest during AO TWs 2–6 (Still Hand period vs: AO TW2 *p* = 0.04; AO TW6 *p* = 0.013; all other *p*s < 0.001).

For the Perspective × Time Window interaction, post hoc tests confirmed that during AO TWs 2–6 for all perspectives, AO induced stronger desynchronization than in all preceding periods of interest (AO TW2 vs Still Hand: LE *p* = 0.008; all other *p*s < 0.001). Furthermore, among these significant TWs, a modulation of beta suppression across perspectives emerged in AO TWs 2 and 4. Beta suppression was stronger for FP and TP than LE (*p* = 0.004, *p* = 0.005, respectively) during AO TW2 and for FP than LA (*p* = 0.035) during AO TW4.

In summary, both significantly stronger central beta suppression during AO relative to preceding periods and significant modulation of beta suppression across perspectives were present in AO TWs 2 and 4 and. Hence, AO TWs 2, 3 and 4 were selected for further analyses.

#### 4 (Perspective) × 2 (Observed Hand) × 2 (Hemisphere) repeated-measures ANOVA

Given the results of the previous ANOVA, to evaluate the effect of the observed hand on central beta desynchronization and the involvement of the two hemispheres in different AO perspectives, we averaged power ratio values over AO TWs 2, 3 and 4.

The repeated-measures ANOVA showed a significant main effect for Perspective (*F*(3,45) = 3.65; *p* = 0.019, η_p_^2^ = 0.196) (Fig. 5b). FP induced stronger central beta suppression than LA (*p* = 0.039) and, with a trend toward significance, LE (*p* = 0.067).

## Discussion

The present study evaluated the reactivity of sensorimotor mu rhythm during observation of right and left hand actions from four perspectives, varying in terms of position of the observer relative to the moving agent (identifying longitudinal and lateral viewpoints) and of anatomical compatibility of observed effectors with self-body parts (identifying ego- and allo-centric viewpoints).

Overall, our results showed not only a significant mu rhythm reactivity during AO for all perspectives, but also a clear view-dependence in the strength of mu desynchronization, in both alpha and beta subcomponents. Concerning the central alpha range, stronger suppression for AO with respect to static visual stimuli for all tested perspectives, as well as differences of mu suppression across viewpoints, occurred between 500–1250 ms from action onset. During this whole period, FP induced stronger modulation than TP and LE, while a trend toward a significantly stronger suppression in FP relative to LA emerged between 500–750 ms after action onset. Taken together, these findings indicated an overall preference of central alpha for the FP.

Our results confirmed the stronger central alpha desynchronization for FP than TP that was reported in most of prior EEG studies^14,15^. In this regard, in the only EEG study reporting greater mu suppression for TP compared to FP AO^13^, the stimuli in the egocentric condition showed not only the actor’s hand and forearm, but also part of the actor’s shoulder and back. As previously suggested^15^, these visual features could have converted the FP in a non-egocentric viewpoint, as if participants observed another agent from his/her back, hence reducing the level of perspective-related reactivity of mu rhythm.

Furthermore, our analyses revealed that FP induced stronger central alpha reactivity also with respect to lateral viewpoints, which so far were not investigated in human research on this topic. Collectively, prior and present data on alpha range point toward a prominent role of FP relative to other perspectives in terms of evoked motor reactivity during AO, due to greater visuospatial congruency between viewed actions and self-action representations in onlooker’s motor system.

It is worth noting that all prior EEG studies focused on alpha mu subcomponent and overlooked modulatory effects of AO viewpoint on the central beta band. Filling this gap in the literature, our data showed, beside the prominent alpha subcomponent reactivity, perspective-dependent responses also in the central beta range, although with lower strength. Furthermore, a different pattern of reactivity with respect to the alpha band emerged. Indeed, for the beta subcomponent, stronger suppression during AO than during preceding visual stimulation for all perspectives, and viewpoint-dependent modulations in the degree of mu suppression, were present between 250–1000 ms from action onset. During this interval, beta reactivity was stronger for FP with respect to lateral perspectives, while no significant differences emerged between FP and TP. Furthermore, stronger beta desynchronization for TP than LE emerged between 250–500 ms from action onset. These divergent results for central alpha and beta bands suggest different functional meanings for perspective-related modulations in these frequency ranges. Interestingly, previous evidence linked the reactivity of central beta, rather than alpha range, to the encoding of kinematic parameters of observed actions, such as velocity profiles^35^. Furthermore, magnetoencephalographic data revealed the tuning of beta rhythm to the dynamic spatial location where observed actions occurred, regardless of which (right or left) arm carried out the movement^36^. In the light of this prior evidence, a hypothetical explanation for our results could be the sensitivity of central beta rhythm to a specific parameter of observed actions, namely the direction in space with respect to the onlooker.

Of note, actions unfolding in a sagittal plane relative to the observer (both away and towards, as in our study in FP and TP, respectively) are suggestive of a face-to-face interaction with another agent, which can implicate a cooperative or competitive behavioural response. During social interactions, both self and others’ actions require detailed coding of kinematic parameters, so that the observer could produce successful behavioural output. Hence, according to our hypothesis, such subjective relevance of actions developing in a sagittal direction relative to the observer, in terms of possible motor interactions with conspecifics, would be reflected in strong beta desynchronization for both FP and TP. Conversely, our reach-to-grasp actions observed from lateral perspectives, unfolding in a parallel plane with respect to the onlooker, would suggest a less stringent possibility of an interaction with an observed agent. Although this speculative interpretation of the perspective-related reactivity of the central beta rhythm needs to be confirmed in future EEG studies, it is interesting to note that a recent fMRI study revealed a specific cerebral network for the processing of observed actions directed in the three-dimensional space toward the observer^37^. During the presentation of videos in which some hand actions unfolded toward participants when shown from TP and others when shown from LE, stronger activation emerged in ventral premotor, parietal and insular regions, for movements toward the observer, in both AO perspectives. Furthermore, the ventral premotor cortex showed a preferential reactivity for TP relative to LE, regardless of the direction of actions in space, suggesting a role for this cerebral region “*in face-to-face interactions*, *requiring accurate assessment of actions performed by conspecifics*, *especially when directed toward the observer*.”^37^.

Another relevant aspect that so far available EEG literature has not taken into account is the effect of lateral perspectives, relative to other viewpoints, on cerebral responses during AO. In this regard, our study revealed a complex pattern of EEG reactivity to the four tested perspectives: in both alpha and beta ranges, the degree of mu suppression was highest for FP, lowest for LE, and at an intermediate level for LA. Furthermore, TP induced different levels of suppression in the two frequency bands. The direct comparison between different longitudinal and lateral perspectives allowed us to highlight new aspects of perspective-related mu rhythm reactivity. On the one hand, while prior EEG data, comparing only FP and TP and analysing only the alpha band, suggested a categorical dichotomy in cerebral processing of egocentric vs allocentric viewpoints, our results revealed a smooth transition in the strength of cerebral responses among FP-lateral-TP viewpoints. These findings are new in humans, but are in line with data in monkeys’ single-cell and LFP recordings of F5 MNs during AO^4,5^. On the other hand, our results are inconsistent with the hypothesis that stronger sensorimotor reactivity to ego- relative to allo-centric viewpoints is ascribable to a coarse morphological and visuospatial congruence with self-effectors. According to this latter hypothesis, on the one hand, both FP and LE, considered “egocentric” since anatomical details of displayed actions are congruent with those of observed self-movements and body parts, should induce the strongest levels of mu suppression. On the contrary, LE evoked significantly lower suppression than FP in both frequency ranges, matching recent single-neuron recording evidence in monkeys of a preferential tuning of F5 MNs to actions seen from FP relative to LE^6^. On the other hand, LE should induce stronger mu suppression than LA, while no significant differences emerged in our study either in alpha or in beta ranges.

Taken together, these data suggest that, beside the morphological compatibility with self-body segments, other parameters of observed actions and effectors allow the stronger matching with self-action representations. Possibly, a critical parameter could be represented by the angle of rotation of the moving effector in the visual field. In this regard, it would be decisive to test mu rhythm modulations induced by progressive increments of the this angle from 0°, corresponding in our study to FP, to 90° or −90°, corresponding to our LE, respectively for RH and for LH. This analysis could define the AO perspective able to induce the strongest sensorimotor reactivity: for hand actions, this viewpoint could be around ±45°, correspondent to the most natural subjective view of our own upper-limb movements. Furthermore, this evaluation could reveal a maximum angle representing a perspective cutoff, beyond which observed actions would be encoded as only congruent with movements performed by another individual and pertaining to allocentric viewpoints, regardless their biomechanical compatibility with observed self-body segments.

Beside the effect of perspective *per se*, the present study also evaluated whether during AO the laterality of the observed hand influenced view-dependent mu reactivity, and whether a hemispheric lateralization of mu suppression occurred. For the alpha range, mu suppression was stronger in the left central cluster of electrodes, irrespective of the observed hand and of AO perspective, while no significant main effect or interactions emerged for the “Observed Hand” factor. Of note, the divergence between this pattern of alpha reactivity at central electrodes and alpha modulations at posterior sites, allowed us to rule out simple effects of volume conduction from posterior to rolandic regions and to confirm the presence during AO of different (although closely coordinated) simultaneous processes in central and occipitoparietal regions (see “EEG Complementary Analyses” in “Supplementary Information”). Nevertheless, our data did not clarify inconsistencies concerning mu lateralization during AO that emerged in prior studies, which reported either bilateral (e.g.,^32,33,35^) and right-lateralized mu suppression, regardless of the side of observed hand^13^, either a contralateral desynchronization for AO of unilateral upper-limb actions^38^. Concerning our results, stronger alpha suppression in left central electrodes might reflect a general engagement of the motor system during AO, in accord with hypothesized left-hemispheric bias for motor control. Among possible alternative explanations for this finding, concomitant movements of participants’ right hand were ruled out by EMG recording, while an asymmetric preparation of motor responses to the attentional task is unlikely, since responses with both hands were required. Nevertheless, since our participants were all right handed, we cannot exclude the role of observers’ handedness on lateralization of mu reactivity, as previously reported for action execution (e.g.,^39^). Furthermore, observer’s hand dominance could also influence perspective-related mu modulations during AO. Indeed, a previous EEG study in right-handed subjects showed greater alpha mu suppression for FP than TP only during movements of the RH, corresponding to observers’ dominant hand, and for right than for left hand only in FP^15^. Possibly, the more ecological display of action stimuli in this latter experiment, with upper-limb actions shown in subjective perspective at about 45°, could explain inconsistencies with our results. Indeed, by setting the FP at 0, we could have hindered evidence of a prevalent desynchronization for the RH, and of a significant interaction between observed hands and AO perspectives.

In conclusion, our study provides new evidence for a privileged role of FP over all other tested AO perspectives in evoking sensorimotor reactivity, confirming previous data concerning the comparison with the TP perspective (although only for central alpha range) and revealing for the first time a FP advantage also relative to lateral perspectives.

From a theoretical perspective, our data are in line with evidence that the strength of motor resonance (and of the subtended MNs’ activity) during AO is related to the level of prior subjective visuomotor experience with observed actions^40,41^. During AO, the simultaneous activation of visual and motor inputs is more frequent for FP than for other viewpoints: indeed, the visual monitoring of self-movements is far more common than the observation of a conspecific while synchronously reproducing his/her movements. Hence, our data suggest the special role of the observation of own movements in shaping the responsiveness of the motor system during AO^42–44^. Whether and how associative sensorimotor learning mechanisms are exactly involved in the phylo- and onto-genesis of the MNS remain intriguing open questions for future research^42–44^.

Noteworthy, our results have also practical implications, since the possibility to recruit the motor system through AO, also when subjects are not able (e.g., after cerebral insults) or not allowed (e.g., during imposed immobilization) to move, is exploited by the so-called AOT rehabilitative interventions. According to our findings, in order to maximize motor recovery of upper-limb motor function, AOT protocols should implement visual stimuli showing hand actions from a FP, able to elicit the strongest sensorimotor reactivity relative to other perspectives.

Nevertheless, to provide conclusive recommendations, some relevant points that could affect the generalisability of the present findings need further clarification. A first limitation of our study is the relatively low sample size. Hence, future research with higher samples is needed to confirm and extend our findings. A second drawback regards the potential influence of ethnicity on the level of mu rhythm reactivity. Indeed, our findings are valid for Caucasian individuals, since in the present study both the actors in the video clips and all the participants were Caucasian. Whether our results hold true also for individuals of different ethnic groups, and whether a race bias^45^ could influence perspective-related mu responses during observation of actions performed by ethnic outgroup or ingroup members, should be assessed in future research. As a third concern, since our study included only hand actions, whether the FP maintains a privileged role for observation of actions performed with other effectors remains a relevant open issue for basic neuroscience, with a potential cascade effect on gait and lower-limb rehabilitative interventions^46,47^. Finally, since our study included only young healthy adult volunteers, a preferential sensorimotor reactivity to FP should be specifically assessed also in pathological conditions and in different age groups, such as in infancy. Indeed, recent behavioural evidence showed that the FP advantage relative to TP is age related, being absent until nine years of age and reaching full development in the late adolescence^48^.

Notwithstanding these open questions that should be addressed in future research, present results provide a starting point for the selection, in future AOT protocols, of action stimuli that could be the most effective in favouring motor recovery, since chosen on the basis of neurophysiological data. From a wider perspective, our data emphasize the advantages of the integration between basic neuroscience and clinical practice, allowing an optimization of therapeutic interventions on the grounds of neurobiological evidence.

## Electronic supplementary material

Supplementary Information

## Data Availability

The data that support the findings of this study are available from the corresponding author upon reasonable request.
