# Supplementary material for: Perspective-dependent reactivity of sensorimotor mu rhythm in alpha and beta ranges during action observation: an EEG study
Source: Sci Rep. 2018 Aug 20;8:12429. doi: 10.1038/s41598-018-30912-w (PMC6102263; doi:10.1038/s41598-018-30912-w)
Supplement: Supplementary file 1 — Supplementary Information [file 41598_2018_30912_MOESM1_ESM.pdf]

Perspective-dependent reactivity of sensorimotor mu rhythm in alpha and beta ranges during action observation: an EEG study

Monica Angelini[1,2,3]*, Maddalena Fabbri-Destro[1], Nicola Francesco Lopomo[2], Massimiliano Gobbo[3], Giacomo Rizzolatti[1], Pietro Avanzini[1]

1 Consiglio Nazionale delle Ricerche (CNR), Istituto di Neuroscienze, Sede di Parma, Italy
2 Dipartimento di Ingegneria dell'Informazione, Università degli Studi di Brescia, Italy
3 Dipartimento di Scienze Cliniche e Sperimentali, Università degli Studi di Brescia, Italy

* Corresponding author
Email: monica.angelini@unibs.it

Supplementary Information

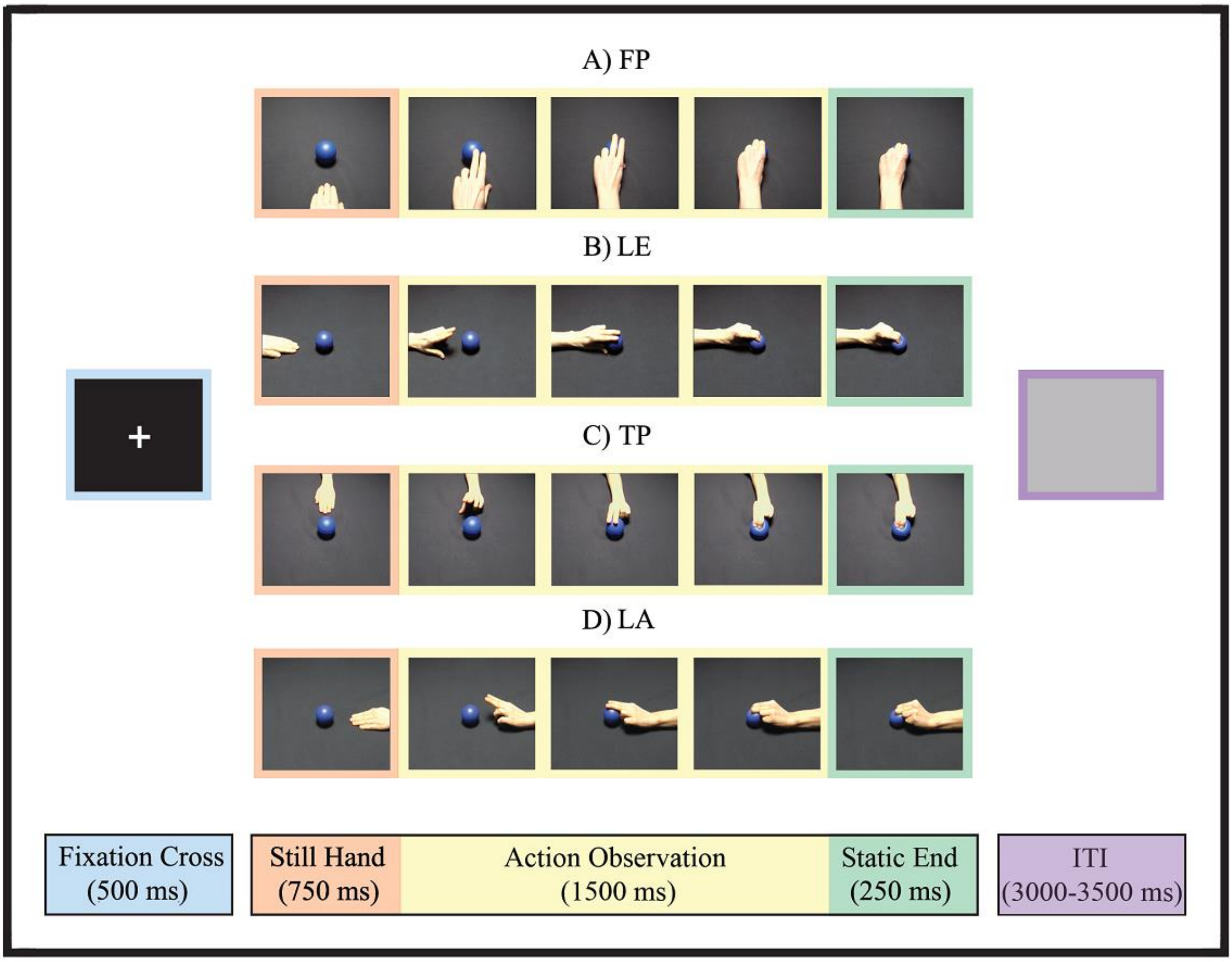

Supplementary Figure S1. Experimental stimuli and structure of the Condition Trials. Trial events, their durations and single frames extracted from video clips of each perspective are shown. In order to provide a further example of our experimental stimuli, the grip movement performed with the left hand by the female actor is displayed, from each of the four perspectives.
FP: first-person perspective; LE: lateral egocentric perspective; TP: third-person perspective; LA: lateral allocentric perspective.

1) EMG Analyses and Results

EMG data corresponding to the analysed EEG epochs were averaged across time during the 1500 ms corresponding to the observation of the movement (AO EMG data), and during the 300 ms of ITI used as baseline for EEG time-frequency analyses (Baseline EMG data), for each participant, condition and right and left FDI. AO EMG data were normalized by division with correspondent Baseline EMG data. After log10 transform, resulting EMG ratio data were tested in: 1) one-tailed single-sample *t* tests against 0 ($p<0.05$), in order to rule out significant stronger EMG activity during AO with respect to the baseline. Log10 EMG ratio values higher than 0 index stronger muscular activity during AO than during the baseline; 2) a repeated-measures ANOVA ($p<0.05$) with Muscle (2 levels: right and left FDI) and Conditions (8 levels: FP-LH, FP-RH, LA-LH, LA-RH, LE-LH, LE-RH, TP-LH, TP-RH) as within-subject factors, in order to rule out significant differences in EMG activity across conditions. In case of violation of the sphericity assumption at Mauchly's test, Greenhouse-Geisser correction was applied, with uncorrected degrees of freedom and corrected *p* values reported.

All *t* tests (right FDI: FP-LH $t(15)=-1.54$, FP-RH $t(15)=-1.49$, LA-LH $t(15)=-1.57$, LA-RH $t(15)=-1.53$, LE-LH $t(15)=-1.35$, LE-RH $t(15)=-1.63$, TP-LH $t(15)=-1.71$, TP-RH $t(15)=-1.14$; left FDI: FP-LH $t(15)=-2.26$, FP-RH $t(15)=-1.74$, LA-LH $t(15)=-2,12$, LA-RH $t(15)=-1.82$, LE-LH $t(15)=-2.06$, LE-RH $t(15)=-1.98$, TP-LH $t(15)=-2.08$, TP-RH $t(15)=-2.06$) and ANOVA's main effects and interaction (Muscle: $F(1,15)=0.5$, $p=0.47$; Condition: $F(7,105)=0.44$, $p=0.87$; Muscle x Condition: $F(7,105)=1.45$, $p=0.23$) were not significant, ruling out that different muscular activity could explain differences in mu rhythm suppression.

2) EEG Preprocessing

Mean number of accepted trials (SD) for each condition was: FP-LH 36.6 (1.5); FP-RH 36.3 (2.1); LA-LH 36.1 (2.1); LA-RH 35.6 (1.7); LE-LH 36.9 (1.7); LE-RH 36.6 (1.3); TP-LH 35.6 (2.2); TP-RH 36.5 (1.8). A one-way repeated-measures ANOVA ($p<0.05$) ruled out differences in the number of accepted trials ($F(7,105)=1.11$, $p=0.36$).

3) EEG Complementary Analyses

Methods

For a comprehensive description of posterior alpha rhythm reactivity and to rule out the influence of volume conduction on central alpha mu subcomponent, the following EEG complementary analyses were performed on power data from occipitoparietal clusters:

a) Single-subject power ratio values were averaged across Observed Hand (LH, RH) and left and right posterior clusters of electrodes, separately for each perspective. Log10 AO ratio data were tested in one-tailed single-sample *t* tests against 0 ($p<0.05$). For each perspective, log10 ratio values significantly lower than 0 indicated significant suppression of posterior alpha during AO relative to the baseline.

b) Ratio data were averaged across Observed Hands and right and left occipitoparietal cluster of electrodes, separately for each Perspective. Log10 ratio values were tested in a repeated-measures ANOVA ($p<0.05$) with Perspective (4 levels: FP, LA, LE, TP) and Time Window (9 levels: ITI, Fixation Cross and Still Hand periods, six TWs during AO) as within-subject factors.

c) Occipitoparietal power ratio were averaged across the same AO TWs that showed at central electrodes a reactivity of alpha mu subcomponent both specific for AO and sensitive to the effect of perspective (see the second step of EEG statistical analyses in "Methods" in the main text). A 4

(Perspective: FP, LA, LE, TP) x 2 (Observed Hand: LH, RH) x 2 (Hemisphere: occipitoparietal left and right clusters of electrodes) repeated-measures ANOVA (p<0.05) was performed on such averaged occipitoparietal log10 ratio data.

For all ANOVAs, in case of significant main effects and/or interactions, Bonferroni-corrected post hoc tests were computed and in case of violation of the sphericity assumption at Mauchly's test, Greenhouse-Geisser correction was applied, with uncorrected degrees of freedom and corrected and significant p values reported.

Results

a) Single-sample t tests

For all perspectives, one-tailed single-sample t tests against 0 resulted significant (FP $t(15)=-15.82$, LA $t(15)=-9.29$, LE $t(15)=-9.88$, TP $t(15)=-10.38$; all ps<0.001). These results proved that during AO all perspectives induced an overall significant suppression in alpha range with respect to the baseline at the occipitoparietal level.

b) 4 (Perspective) x 9 (Time Window) repeated-measures ANOVA

The 4 (Perspective) x 9 (Time Window) repeated-measures ANOVA showed significant main effect of Perspective ($F(3,45)=4.21$, $p=0.01$, $\eta_p^2=0.219$) and Time Window ($F(8,120)=78.98$, $p<0.001$, $\eta_p^2=0.84$,) and a significant Perspective x Time Window interaction ($F(24,360)=4.84$, $p<0.001$, $\eta_p^2=0.244$) (Supplementary Fig. S2a).

For the main effect of Perspective, post hoc tests showed an overall stronger suppression for FP than TP ($p=0.007$). For the main effect of Time Window, post hoc tests showed stronger suppression relative to all preceding visual stimuli only for AO TW4 (AO TW4 vs Still Hand: $p=0.01$; all other ps <0.001).

The significant Perspective x Time Window interaction modulated both main effects. Bonferroni-corrected post hoc tests for the Perspective x Time Window interaction confirmed stronger desynchronization during AO than during all preceding periods of interest for all perspectives in AO TWs 4 and 5 (AO TW4 vs Still Hand: TP $p=0.002$; AO TW5 vs Still Hand: FP $p=0.007$, TP $p=0.001$; all others ps<0.001). Within these significant TWs, only AO TW4 showed a significant modulation of suppression across Perspectives, with both FP and LA inducing stronger desynchronization than TP ($p<0.001$, $p=0.02$, respectively).

c) 4 (Perspective) x 2 (Observed Hand) x 2 (Hemisphere) repeated-measures ANOVA

The 4 (Perspective) x 2 (Observed Hand) x 2 (Hemisphere) repeated-measures ANOVA (performed on occipitoparietal data averaged over AO TWs 3, 4 and 5, which were selected and analysed at central electrodes) showed significant main effect of Perspective ($F(3,45)=6.22$, $p=0.001$, $\eta_p^2=0.293$) (Supplementary Fig. S2b) and Perspective x Hemisphere x Hand interaction ($F(1,15)=6.92$, $p<0.001$, $\eta_p^2=0.316$) (Supplementary Fig. S2c).

Overall, FP induce stronger suppression than TP ($p<0.001$). At post hoc tests for the significant interaction, stronger suppression in right than in left occipitoparietal cluster emerged during observation of both hands for FP and TP (right vs left hemisphere: FP-LH $p=0.012$, FP-RH $p<0.001$, TP-LH $p<0.001$, TP-RH $p=0.026$), and only for observation of a RH from LA and of a LH from LE (right vs left hemisphere: LA-RH $p=0.0014$, LE-LH $p<0.001$). Comparing the suppression during observation of a RH vs LH, no significant differences emerged for FP and TP in both posterior clusters, and in left posterior cluster for both lateral perspectives. Conversely, in right occipitoparietal cluster, alpha suppression was stronger for observation of a RH than of a LH for LA ($p=0.006$), and of a LH than of a RH hand for LE ($p=0.007$). In both posterior clusters, stronger suppression for FP than TP (all ps<0.001) was present for AO of both hands. For lateral perspectives, in right posterior cluster stronger desynchronization was induced by observation of a LH from LE than LA ($p=0.044$), and of a RH from LA than LE ($p<0.001$).

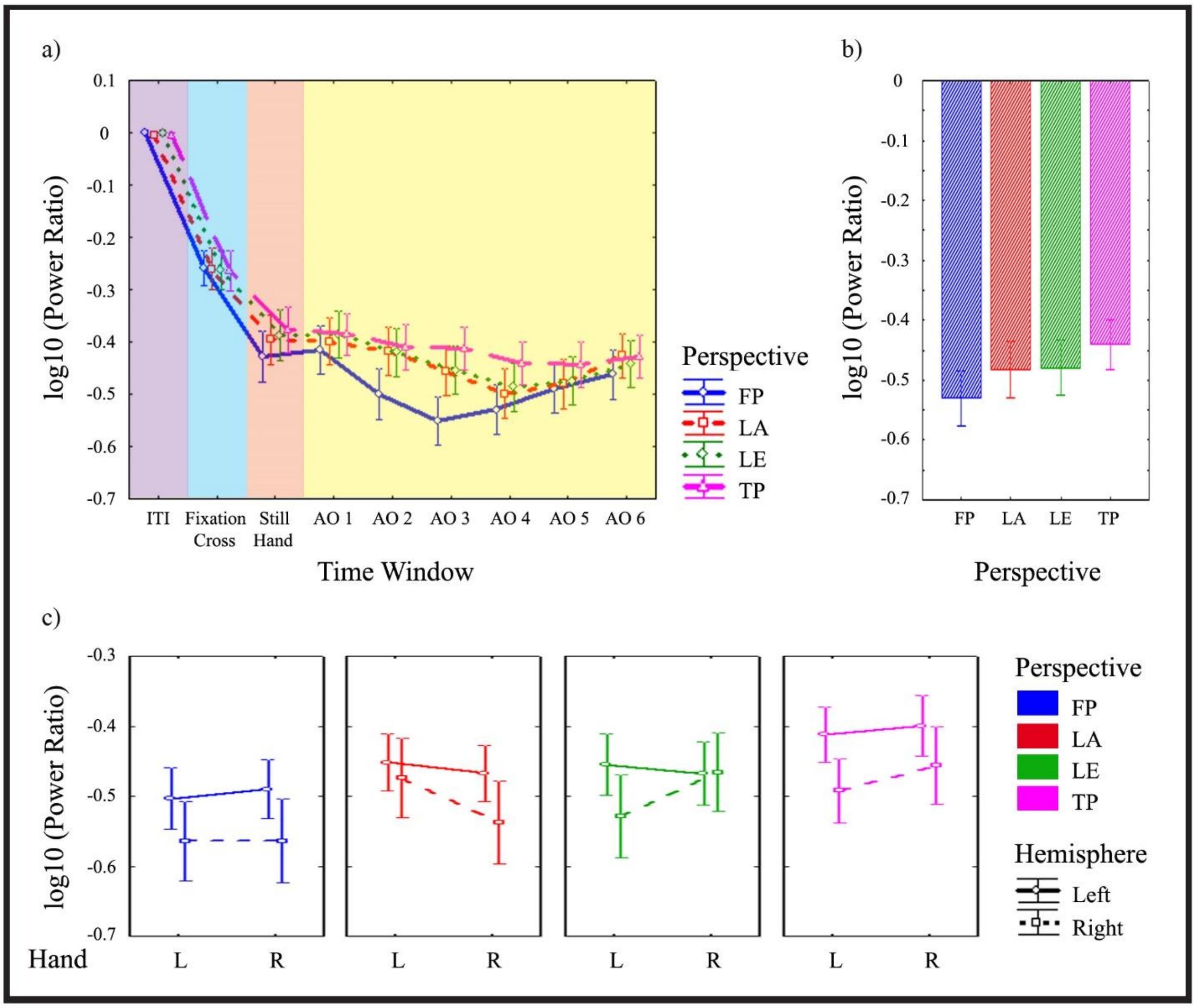

Supplementary Figure S2. Results of EEG complementary analyses on power ratio data in alpha (8-13 Hz) frequency range from occipitoparietal clusters of electrodes. a) Time course of posterior alpha (8-13 Hz) log10 power ratio for each perspective, averaged across right and left occipitoparietal clusters of electrodes and left and right observed moving hands. Each time window within the epoch (ITI, Fixation Cross, Still Hand presentation periods, six Action Observation time windows of 250 ms each) is labelled by different colours. b) Log10 power ratio of posterior alpha frequency range for each perspective, averaged during the time interval analysed for central alpha (action observation time windows 3, 4, 5). The figure represents the significant main effect of Perspective in the 4 (Perspective) x 2 (Observed Hand) x 2 (Hemisphere) repeated-measures ANOVA performed on occipitoparietal log10 power ratio in the alpha range. c) Log10 power ratio of posterior alpha for each Perspective, Observed Hand and Hemisphere during action observation time windows 3, 4, 5. The figure represents the significant Perspective x Observed Hand x Hemisphere interaction in the 4 (Perspective) x 2 (Observed Hand) x 2 (Hemisphere) repeated-measures ANOVA performed on occipitoparietal power ratio in the alpha range. Error bars represent the standard errors of the mean. FP: first-person perspective; LE: lateral egocentric perspective; TP: third-person perspective; LA: lateral allocentric perspective; ITI: intertrial interval period; AO: Action Observation time window.

In summary, EEG complementary analyses revealed relevant differences between alpha modulation at central and occipitoparietal cluster of electrodes (summarized in Supplementary Table S1), ruling out the role of simple volume conduction from posterior sites on central mu rhythm.

Supplementary Table S1. Summary of EEG analyses results in alpha range (8-13 Hz) at central and occipitoparietal clusters of electrodes.

	Central cluster	*Occipitoparietal cluster*
ANOVA 4 (Perspective) x 9 (TW)		
- Main Effect of Perspective	FP > LE	FP > TP
- Main Effect of TW	AO TWs 3-5 > I, F, S	AO TW4 > I, F, S
- Perspective x TW interaction:		
1) suppression in AO TW > I, F and S for all perspectives (defying significant AO TWs)	AO TWs 3,4,5	AO TWs 4, 5
2) suppression modulation across perspectives in significant AO TWs	AO TW3: FP > LE, TP<br>AO TWs 4, 5: FP > TP	AO TW4: FP, LA > TP
ANOVA 4 (Perspective) x 2 (Observed Hand) x 2 (Hemisphere) in AO TWs 3-5		
- Main effect of Perspective	FP > LE, TP	FP > TP
- Main effect of Hemisphere	Left > Right	ns
- Perspective x Hand x Hemisphere interaction	ns	p<0.001

TW: time window; AO: action observation; I: intertrial interval period; F: fixation cross period; S: still hand period; FP: first-person perspective; LA: lateral allocentric perspective; LE: lateral egocentric perspective; TP: third-person perspective; ns: not significant.

Discussion

In our study, perspective-related EEG modulations in alpha range emerged at both central and occipitoparietal clusters of electrodes, but with different patterns of reactivity (Supplementary Table S1). These findings are in line with the hypothesis that central mu and occipital alpha rhythms reflect different but highly coordinated processes during AO, indexing a close interplay between sensorimotor resonance and visuospatial and attentional processes. With regard to the comparison between FP and TP, in our study an overall stronger suppression for the former emerged bilaterally at occipitoparietal level, matching previous EEG and fMRI evidence. Indeed, Drew *et al.* reported stronger alpha suppression in right occipital electrodes during observation of RH actions from FP relative to TP[15], while a prior fMRI experiment[10] found increased activity of bilateral cuneus during observation of intransitive right hand and foot actions from FP compared with TP. Notably, a lateralized location of action stimuli in one visual hemifield, with consequent contralateral occipitoparietal activation during allocation of visuospatial attention, cannot account for occipital perspective-dependent different responses in these latter experiments. Indeed, in the above-mentioned EEG study[15], perspective effect was only present for RH and not for LH, and was lateralized to ipsilateral right occipital electrodes, while in the latter fMRI study[10], actions were displayed around the midline of the screen, and stronger responses for FP emerged in bilateral occipital cortices. Interestingly, preferential responses to FP relative to TP in bilateral occipital areas have been also reported by a previous PET study[49] comparing a motor imagery (MI) task requested from FP and TP perspectives. These latter findings proved that not only actual motion perception, but also pure internal rehearsal of motor representations are able to elicit stronger responses in occipital regions for FP. Taken together, present and previous data suggest an intrinsic preferential tuning of cerebral visuospatial and attentional processing to FP relative to TP during covert action simulations, both voluntarily triggered (as during MI) and automatically evoked (as during AO).

Another interesting finding in our study concerns the lateralization of posterior alpha suppression. Significant stronger alpha suppression on right occipitoparietal cluster of electrodes was present,

regardless of the observed moving hand, for actions seen from both FP and TP, which unfolded longitudinally along the visual field midline. Conversely, for lateral perspectives, stronger suppression in right posterior cluster was present only for action stimuli that were presented in the left part of the screen, namely for observation of a moving RH from LA and of a moving LH from LE. For action stimuli presented in the right part of the screen, an equal bilateral level of alpha desynchronization was present at occipitoparietal level. Furthermore, within each lateral perspective, the comparison between right and left moving hands showed stronger desynchronization in right posterior cluster for RH vs LH from LA and for LH vs RH from LE. Collectively, this pattern of results is in accordance with the hypothesis of a right hemispheric dominance for visuospatial and attentional processing, and of an asymmetric distribution of visuospatial attention along horizontal axis of the visual field, producing the leftward attentional bias reported in young adults called "pseudoneglect"[50]. Since an accurate evaluation of visuospatial and attentional processing reflected in the posterior alpha rhythm is outside the aims of the present study, this aspect was not further addressed.

In conclusion, our data on alpha frequency range suggest a close interaction between visuospatial and sensorimotor processing during observation of actions from different perspectives. Notably, reciprocal interactions between occipital and motor areas include not only a feedforward flow of information that can favour sensorimotor resonance during AO, but also backward, top-down connections that could regulate visual perception of actions[51]. Indeed, it has been proposed that a relevant function of perspective-dependent F5 MNs in monkeys is a top-down modulation of high-level visual areas through backward projections, reinforcing the perception of specific details associated with different views of the same action[4]. Hence, future experiments should aim at assessing how the bidirectional flow of information between occipitoparietal and parietocentral cortical regions influences both attentional and sensorimotor activity during AO.

Supplementary References

49. Ruby, P. & Decety, J. Effect of subjective perspective taking during simulation of action: a PET investigation of agency. *Nat Neurosci*. **4,** 546-550 (2001).
50. Brooks, J.L., Della Sala, S. & Darling, S. Representational pseudoneglect: a review. *Neuropsychol Rev*. **24,** 148-165 (2014).
51. Avanzini, P. *et al*. Spatiotemporal dynamics in understanding hand-object interactions. *Proc Natl Acad Sci USA*. **110,** 15878-15885 (2013).
